# Gridded global datasets for Gross Domestic Product and Human Development Index over 1990–2015

**DOI:** 10.1038/sdata.2018.4

**Published:** 2018-02-06

**Authors:** Matti Kummu, Maija Taka, Joseph H. A. Guillaume

**Affiliations:** 1 Water & Development Research Group, Aalto University, Tietotie 1E, 02150 Espoo, Finland

**Keywords:** Economics, Developing world, Environmental social sciences

## Abstract

An increasing amount of high-resolution global spatial data are available, and used for various assessments. However, key economic and human development indicators are still mainly provided only at national level, and downscaled by users for gridded spatial analyses. Instead, it would be beneficial to adopt data for sub-national administrative units where available, supplemented by national data where necessary. To this end, we present gap-filled multiannual datasets in gridded form for Gross Domestic Product (GDP) and Human Development Index (HDI). To provide a consistent product over time and space, the sub-national data were only used indirectly, scaling the reported national value and thus, remaining representative of the official statistics. This resulted in annual gridded datasets for GDP per capita (PPP), total GDP (PPP), and HDI, for the whole world at 5 arc-min resolution for the 25-year period of 1990–2015. Additionally, total GDP (PPP) is provided with 30 arc-sec resolution for three time steps (1990, 2000, 2015).

## Background & Summary

A growing number of openly available global gridded datasets are providing an increasing variety of opportunities for spatial analyses. Simultaneously, the spatial precision of datasets is also increasing. For example, the latest population dataset provides global population count at 250 m resolution^1^, and annual global forest loss data is available with 30 m resolution^2^. Additionally, good data coverage exists for various fields, such as earth science^2–6^, population dynamics^1,7,8^, natural hazards^9,10^ and agriculture^11–13^. This advancement has supported current research across the disciplines^14–20^.

At the same time, however, most of the human and economic development indicators are still provided mainly at national level when presented at global scale, for example by large institutes such as the World Bank and United Nations Development Programme (UNDP). When used for gridded analyses, the nationally reported values are often distributed spatially. For example, Gross Domestic Product (GDP) is distributed according to population density, effectively using the same per capita value across the country^21–24^, or in some cases differentiating between urban and rural areas^9,25^. Realistic global gridded human and economic development indicators are still in their infancy^26,27^, but the existing sub-national data for many countries^28^ has potential for wide use. Openly available gridded datasets for these indicators could help shift the baseline to using data from sub-national rather than national administrative units, particularly when performing analyses in combination with other available global datasets, often with higher precision than country scale.

Two global key indicators of development are Gross Domestic Product (GDP) and Human Development Index (HDI). While ‘GDP measures the monetary value of final goods and services—that is, those that are bought by the final user—produced in a [given area] in a given period of time’^29^, HDI is a composite index of ‘average achievement in key dimensions of human development: [i.] a long and healthy life, [ii.] being knowledgeable and [iii.] have a decent standard of living’^30^. These two indices are often used by various international organisations to describe the development status of an area, and are thus important to include in analyses.

While a dataset is available from UNEP/GRID-Geneva providing global gridded total GDP^9^, it is not based on openly available population data. Thus, data on GDP per capita, which is for many applications a more useful indicator, are not available for users. Additionally, the data represent only one year (2010). There is, however, a sub-national GDP per capita dataset compiled by Gennaioli, *et al.*^28^ that covers a time span of 60 years (1950–2010), but these data are currently only in tabulated format. More broadly, the estimation of GDP density (gridded GDP per unit area) has also been an active area of research, e.g., using luminosity and other physical attributes in addition to existing national and subnational datasets^26,27^. The G-Econ dataset^27,31^ is provided at 1 degree resolution for the years 1990, 1995, 2000 and 2005. Even though our new product cannot be used as a replacement for the G-Econ dataset, it provides more recent and frequent data with no missing data areas, using a simpler methodology and a more restricted set of data sources (limited to national and sub-national administrative units), treating the official statistics as the gold standard.

In this paper, we present altogether three gridded global datasets, all for the years 1990–2015 (see summary in Table 1; note: the first two datasets represent the average value of a parameter in question in a given administrative unit): i) GDP per capita (PPP, i.e. purchasing power parity), ii) HDI. We then used GDP per capita (PPP) and the gridded population dataset HYDE 3.2 to derive iii) the total GDP (PPP) for each grid cell.

The developed datasets make use of the available sub-national data whenever possible, combined with national data. We base our sub-national GDP data on the above mentioned article by Gennaioli, *et al.*^28^, while for the national level data, we used the latest World Bank dataset^32^ supported by data from CIA’s World Factbook^33^. In this study, the sub-national data for HDI were collected from various national-level datasets i) *outside Europe*: censuses and UNDP reports, and ii) *within Europe*: Eurostat database at NUTS (i.e., Nomenclature of territorial units for statistics) level. National level HDI was collected from UNDP^30^. We used the sub-national data to scale the coherent national data, in order to keep them as representative as possible of the official national statistics.

Hitherto, various global studies use the included development indicators in their analysis, including integrated modelling tools^21,22,25^, spatial analyses^34–36^ and hazard exposure and vulnerability assessments^10,23,37,38^. The tradition of using national values is problematic since both GDP and HDI have considerable intra-national variation, emphasized in large countries such as Brazil, China, India, Russia, United States. This new dataset provides a gridded product reflecting best available data at sub-national level where available. Moreover, it provides a data-gap free annual product over the period of 1990–2015. It thus has potential to enhance the accuracy of these global studies, as it more accurately takes into account both spatial sub-country variation and temporal change of these indicators.

## Methods

**Figure 1:**
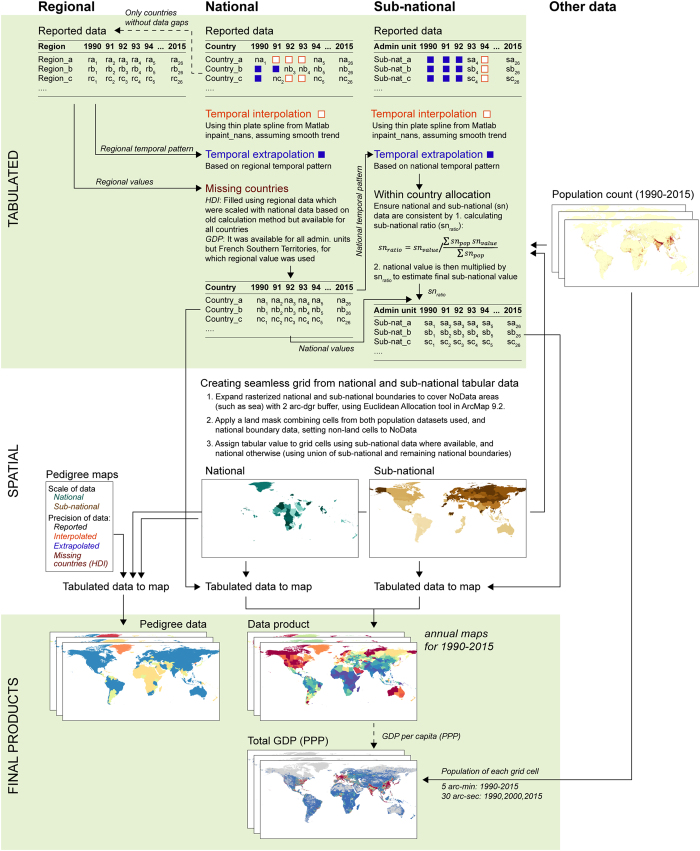
Schematic outline of the methods in creating the gridded products. A similar approach was used for both Gross Domestic Production (GDP) and Human Development Index (HDI). Small differences between the methods between these two data products are noted in the outline.

In this section, we describe in detail how each dataset was produced, including the data sources and assumptions made, where applicable. Overview of the methods is given in Fig. 1.

### Seamless raster grid from national and sub-national administrative datasets

To ensure that our gridded end-products cover the entire land area included in the input datasets, we created a seamless administrative raster file using national (and in some cases also autonomous areas such as Greenland) and sub-national boundaries, and two population datasets (see Table 2). We first expanded the rasterized national and sub-national boundaries to cover NoData areas (such as sea areas and other water bodies) with a 2 arc-degree buffer, using the Euclidean Allocation tool in ArcMap 9.2. Next, we created a land mask by combining cells from both population datasets used, and national boundary data. This land mask was then used to set non-land cells to NoData.

### GDP per capita (PPP)

To compile the GDP per capita (PPP) dataset (Data Citation 1), we first put together a full national GDP dataset, drawn from the most recent World Bank Development Indicators database^32^. For missing countries (see Supplementary Information), we used data from the CIA’s World Factbook^33^, except for one administrative unit, namely French Southern Territories, for which no data was found and regional average data was used (see below). The base year for international US dollars in CIA’s World Factbook was different from those reported by World Bank (Table 2). The constant 2015 international US dollars of CIA fact sheets were converted to constant 2011 international US dollars, the unit in which national GDP from World Bank was given. For that we used the standard method documented by the World Bank^39^.

Temporal coverage of the national data varied considerably between the countries, and thus, to fill the missing values, temporal interpolation and extrapolation approaches were used. For temporal interpolation (i.e., missing years between reported years) we used a thin plate spline to provide a smooth trend over time. This was conducted with the default method of the ‘inpaint_nans’ Matlab package^40^. For temporal extrapolation (i.e., data were missing at either end of the study period; see pedigree data), we used the GDP trend either from other datasets, neighbouring/former countries, or regional data depending on case-specific characteristics:

- for five countries (Haiti, Libya, Maldives, Qatar, Sao Tome and Principe) we used country specific trends from CIA Factbook^33^ to extrapolate World Bank Data^32^, while for Réunion we used Eurostat data^41^ (see Supplementary Information).
- for South Sudan (years 1990–2007) we used trends from Sudan; for Baltic countries (1990–1994) we used trends from Belorussia; and for former Yugoslavian states (1990–1994) we used reported Yugoslavian GDP trend to produce data for missing years.
- for the rest of the countries (for which no data in CIA Factbook^33^ exist nor logical neighbour countries to which to relate the trend) the data was extrapolated over time by scaling the last available value to reflect subsequent regional GDP^32^ changes. Similarly, the first value was scaled with preceding changes (in the same way equation (1), used for sub-national data). Regional data was compiled from reported GDP per capita (PPP) values of countries for which full temporal data coverage exist (*n*=149) which were weighted with population of a given year. We used 12 regions based on the UN classification^42^, and modified by Kummu, *et al.*^43^.

The temporal coverage of tabulated sub-national data was also heterogeneous and we needed to use interpolation and extrapolation, similarly to the national data. Temporal interpolation followed the same method as for national values. The data were extrapolated over time by scaling the last available value to reflect subsequent national changes, and similarly scaling the first value with preceding changes. Equation (1) represents a case when data are missing from the beginning of time series.

(1)snvaluei−1=nvaluei−1nvaluei×snvaluei

where sn_value i-1_ is the first missing sub-national value for time step i−1, n_value i-1_ is reported national value for time step i−1, n_value i_ is the reported national value for time step i, and sn_value i_ is the first reported sub-national value.

To be consistent between the sub-national and national datasets, and to ensure that our dataset would represent official national statistics, we did not use the sub-national GDP per capita (PPP) directly (Table 2) but instead used the sub-national data to scale national GDP per capita (PPP). This was done for each year by first calculating population-weighted national GDP per capita (PPP) from sub-national GDP per capita (PPP) data and the HYDE 3.2 population dataset. This output was then used in each sub-national unit to calculate the ratio between population-weighted national GDP and reported sub-national GDP (see equation (2)). The final sub-national GDP per capita (PPP) was calculated by multiplying the ratio with the reported national GDP per capita (PPP) (equation (3)).

(2)snratio=snvalue/∑snpopsnvalue∑snpop

(3)snfinalvalue=snratio×nvalue

where sn_ratio_ is sub-national ratio, sn_value_ is the reported/estimated sub-national value, sn_pop_ is sub-national population, sn_final value_ is the sub-national value used for final product, and n_value_ is the reported/estimated national value.

After deriving both national and sub-national tabulated data, we first created a raster dataset of these two spatial scales for each time step, and then combined the two raster datasets, which resulted in the final GDP per capita (PPP) raster dataset provided here. Reported sub-national data were used preferentially, followed by interpolated and extrapolated sub-national data, together with national averages. For each year of data, we report the source of the data and, where applicable, the method used to fill the gaps (see Technical Validation for more information).

### Total GDP (PPP)

To estimate the total GDP (PPP) of each grid cell, we multiplied the GDP per capita (PPP) by grid specific population data using two different spatial resolutions: 5 arc-min (10 km at equator) (Data Citation 1) and 30 arc-sec (1 km at equator) (Data Citation 1). Lower resolution population data were taken from HYDE 3.2 which includes annual data for the year 1990 and for the years 2000–2015 with a resolution of 5 arc-min. Thus, for the years 1991–1999 we needed to use interpolated population between 1990 and 2000. Higher resolution population data were adapted from the Global Human Settlement (GHS) population grid^1^ which has data for the years 1990, 2000 and 2015 with a resolution of 30 arc-sec (Table 2).

### HDI

To compile the HDI dataset (Data Citation 1), we first produced a full national HDI dataset, based on the data from the Human Development Reports by UNDP^30^ (Table 3). For non-UN member countries, no up-to-date HDI data exist and we thus used either independent data (Macau, Taiwan; see Supplementary Information) or scaled regional data. To scale the regional data for missing countries we used a near-complete global dataset for year 2009^44^, based on an old HDI calculation methodology. This data set covered all the missing countries except West Sahara for which we used the regional average without scaling. We first calculated the regional average from these data using countries for which full data coverage exists (*n*=144) in the national dataset^29^. This was then used to calculate the scaling factor, in relation to regional data for missing countries (similar to equation (2)), which was then applied similarly to equation (3). We used 12 regions based on UN classification^42^, and modified by Kummu, *et al.*^43^. The countries affected by this assumption are marked as such in the dataset.

The national HDI dataset from UNDP includes all the years 1990–2015. In HDI, no interpolation was needed, as there were no gaps in the data. Data points were only missing from the beginning of the timeseries. In those cases, the data was extrapolated over time by scaling the last available value to reflect subsequent regional changes, and similarly scaling the first value with preceding changes (see equation (1), which represents extrapolation for sub-national data, for which the same method was used).

As to our knowledge no ready database for sub-national HDI data exists, we compiled a new database originating from multiple sources (see details in Supplementary Information). These sources contained data for altogether 39 countries, based on Eurostat^41^ (22 countries) and outside Europe, mostly based on UNDP national surveys and data collected by national statistics offices (see Supplementary Information).

To be consistent between the sub-national and national datasets, and to ensure that our dataset would represent official national statistics, we did not use the sub-national HDI directly (Table 3, Supplementary Information), instead we used the sub-national data to scale the national level HDI. This is important, as in some cases slightly different methods were used for sub-national HDI estimates than for national HDI calculations. The scaling was completed by first calculating population weighted national HDI from sub-national HDI and the HYDE 3.2 population dataset^7^. This was then used in each sub-national unit to calculate the ratio between population-weighted national HDI and reported sub-national HDI (see equation (2)). The final sub-national HDI was calculated by multiplying the ratio with the reported national HDI (equation (3)).

It is important to note that the same temporal ratio (around year 2010; see year of sub-national data in Supplementary Information) was used for all timesteps, as for most of the countries sub-national HDI was available for only one timestep.

After deriving both national and sub-national tabulated data, we first created raster datasets of these two scales for each time step, and then combined the two rasters, resulting in the final HDI raster dataset provided here. Reported sub-national data were used preferentially, followed by interpolated and extrapolated sub-national data, and national averages. For each year of data, we report the source of the data and, where applicable, the method used to fill the gaps (see Technical Validation for more information).

### Error estimation for interpolation and extrapolation

To estimate the error originating from the interpolation and extrapolation to fill the missing data entries, we performed an error analysis separately for national and sub-national level data. For both, we selected the countries that have full temporal data coverage. In interpolation, the main error source is the interpolation method, i.e., thin plate spline, that provides a smooth trend over time between observed data points. Extrapolation, in turn, is based on the temporal pattern of a higher level administrative unit, and error comes from the difference between country specific and regional patterns, or the difference between sub-national and country scale patterns, depending on the scale.

In the analysis, we quantified the agreement between observed and interpolated values $\left(\frac{\left|est-obs\right|}{obs}\right)$ as a function of distance from the nearest time step for interpolation and extrapolation. In interpolation, we applied the thin plate spline interpolation over various lengths of missing data (*n*=1–21) and varied the location of this ‘hole’ over the study period. Next, we collected the relative errors of each entry and grouped them in relation to the distance of an entry to closest observed value, i.e., grouping all points one time step from the nearest observed values, all points two time steps, etc. We performed a similar kind of analysis for estimating the performance of extrapolation, but leaving data entries out from the beginning of the study period, with varying lengths of the missing data (*n*=1–23). Again, we collected the relative error in relation to the distance to the closest observed value. These errors were then plotted together with a 95% confidence interval and further, the maximum errors in each administrative unit were mapped separately for interpolation and extrapolation. Results are reported in the technical validation section, and provided as Supplementary Information to the article.

### Code availability

The creation of datasets was done with Matlab R2016b and code is available at Data Citation 1. Due to copyright issues, we cannot openly share all the input data to run the code, but that data is available on request from the corresponding author.

## Data Records

The datasets are global (180°E–180°W; 90°S–90°N) with a resolution of 5 arc-min (around 10 km at the equator) in standard WGS84 coordinate system. Total GDP (PPP) is also provided at 30 arc-sec for selected time steps. The data are provided in NetCDF-4 format, where the third dimension represents the time step. For each of the four datasets a separate NetCDF-4 file was created (Table 4).

All GDP (PPP) datasets are given in constant 2011 international US dollars for all years within the study period (1990–2015) thus enabling comparison between years. However, when comparing the data, the differing pedigree of the data as well as ecological fallacy may hinder comparability, as is the case with other existing datasets. HDI is a dimensionless indicator scaled between 0 and 1.

### GDP per capita (PPP)

**Figure 2:**
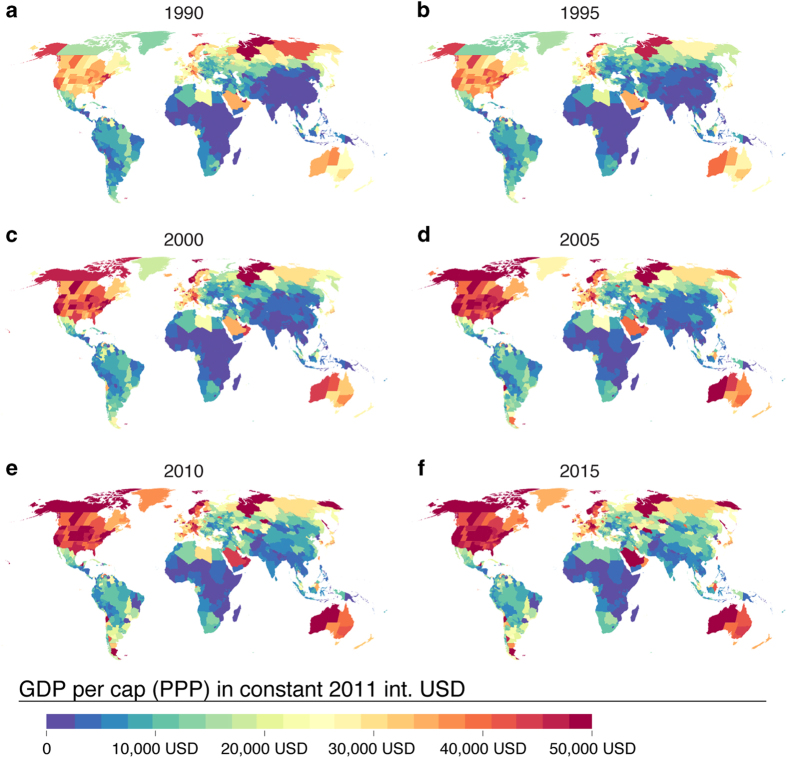
Maps of gridded Gross Domestic Production (GDP) per capita (PPP) in constant 2011 international US dollars (USD) for six selected years over the study period of 1990–2015. Derived from a combination of sub-national and national data (see pedigree map in Fig. 5).

The GDP per capita (PPP) dataset represents average gross domestic production per capita in a given administrative area unit (Fig. 2; see also provided dataset of administrative units). In 1990, the highest GDP per capita areas were found in the US east coast, Northern Siberia, Central and Northern Europe and the Middle East. By the end of the study period (2010–2015), Australia, almost the entire North America and a few places in Central Asia and Southern tip of South America joined these very high (>40,000 USD) GDP per capita areas. Sub-national data clearly show the heterogeneity of GDP per capita development in large countries, such as US, China, Russia and India (Fig. 2).

### GDP (PPP)

**Figure 3:**
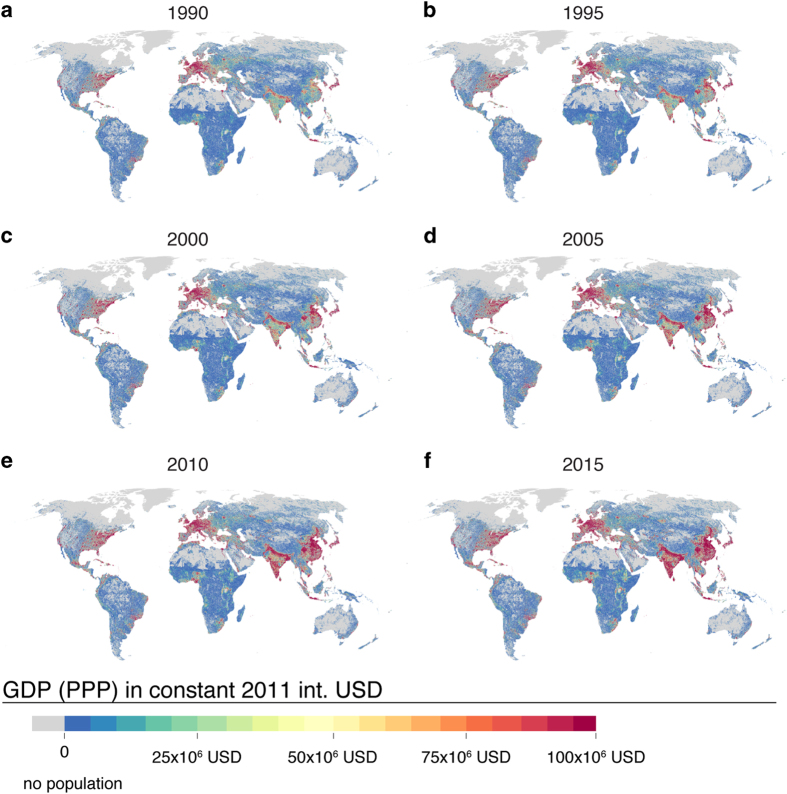
Maps of gridded total Gross Domestic Production (GDP) (PPP) in constant 2011 international US dollars for six selected years over the study period of 1990–2015. Derived from GDP per capita (PPP), which is multiplied by population based on HYDE 3.2.

GDP (PPP) represents total gross domestic production in a given grid cell in constant 2011 international US dollars. Maps for the selected years are presented in Fig. 3. As a result of high population densities combined with high GDP per capita, Europe and the US east coast stand out as high GDP areas (cf. Fig. 2) while highly populated areas with smaller GDP such as the Ganges valley and north-eastern China stand out as moderate-low GDP areas. Rapid growth is visible for both GDP per capita and population in Asia when data for the years 1990 and 2015 are compared (Fig. 3).

### HDI

**Figure 4:**
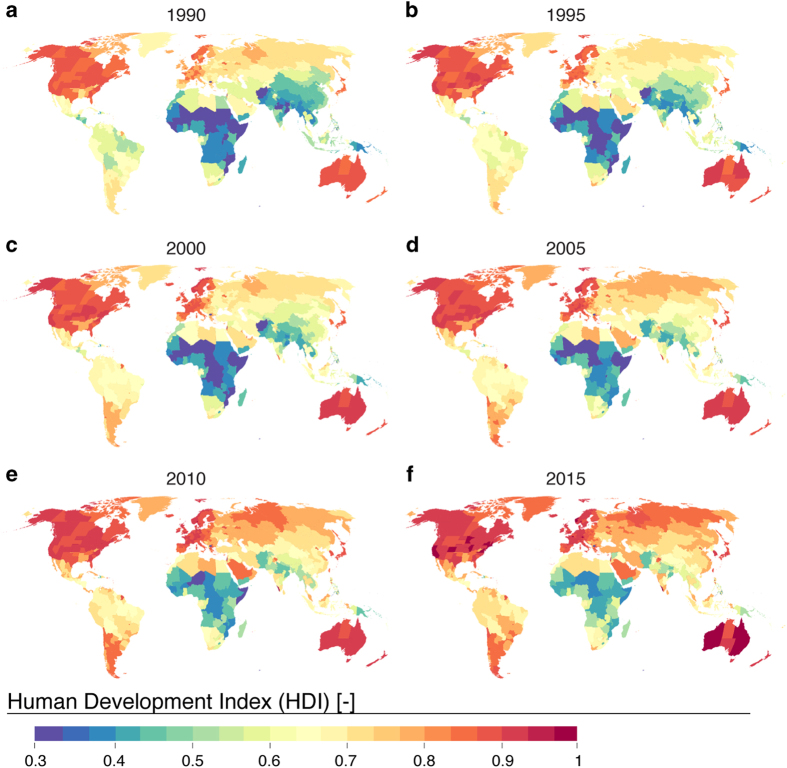
Maps of Human Development Index (HDI) for six selected years over the study period of 1990–2015. Derived from a combination of sub-national and national data (see pedigree map in Fig. 6).

Human Development Index data represent the sub-national data on key aspects of development, namely education, economy and health. Areas of high HDI have been high throughout the study period in North America, Europe, Japan and Australia (Fig. 4). HDI increased remarkably over time in the southern part of South America, Middle East, large parts of Asia and Central Asia.

Sub-national HDI data enable new insights into the heterogeneity of extensive countries, such as US, China, Russia and India (Fig. 4). In China for example, the coastal area has a much higher HDI than the inland provinces, whereas in India, the southwest part has a considerably higher HDI compared to the northern parts of the country.

## Technical Validation

**Figure 5:**
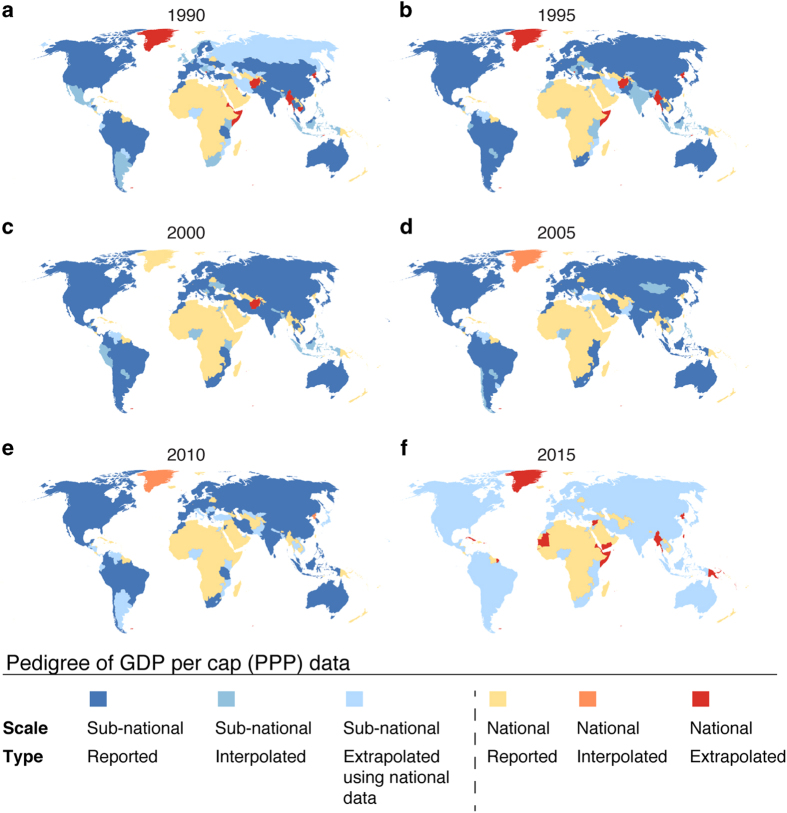
Pedigree maps of Gross Domestic Production (GDP) per capita (PPP) dataset. Pedigree presented in terms of scale of original data source and type of data (reported, interpolated or extrapolated).

**Figure 6:**
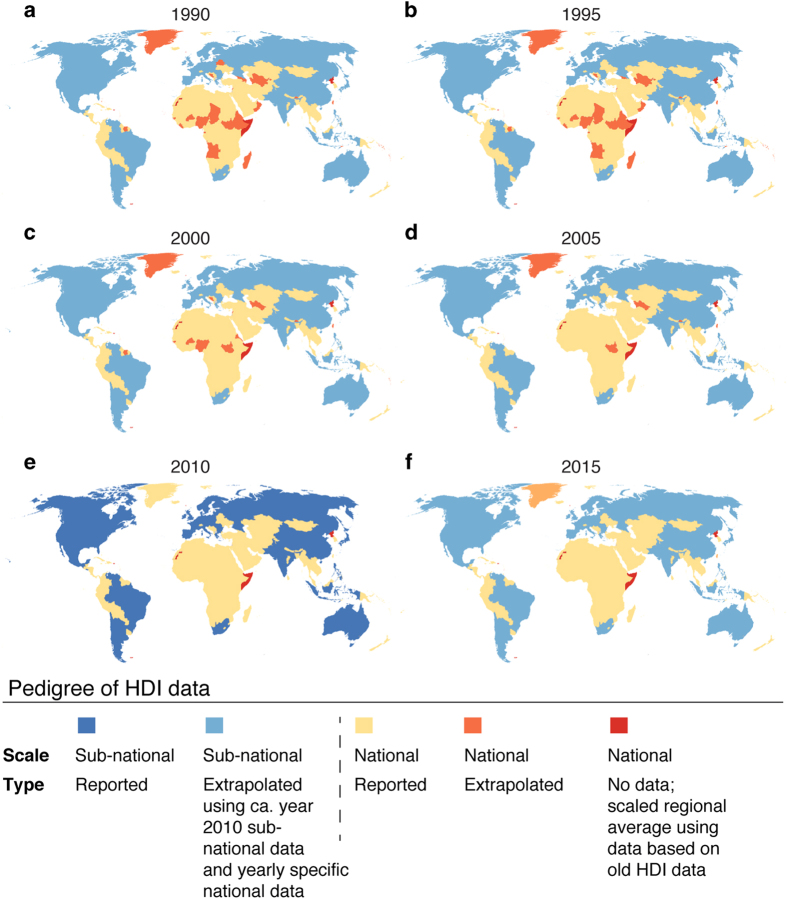
Pedigree maps of Human Development Index (HDI) dataset. Pedigree presented in terms of scale of original data source and type of data (reported, scaled, interpolated or extrapolated).

To make the pedigree of the datasets transparent, we compiled the source of the data for each year and separately for GDP per capita (PPP) (Data Citation 1) and HDI (Data Citation 1). The data are provided in separate NetCDF-4 files (Table 4). The pedigree maps (see Fig. 5 for GDP; and Fig. 6 for HDI) describe the level of input data (sub-national, national) and whether the time step in question is based on reported data, or interpolated/extrapolated data. We also provide the underlying administrative units as a raster dataset, for both GDP per capita (PPP) and HDI (Data Citation 1). The pedigree can be seen as an indication of accuracy and precision. Relative to a particular grid cell, nationally or regionally derived values would generally be considered more uncertain than sub-national values. The nominal value is in general a less accurate representation of the grid value, and should therefore be considered a less precise estimate. Extrapolation and interpolation also entail a loss of accuracy, and the source data may be more or less accurate, as our error analysis indicates. The effects of these issues are difficult to quantify, such that their interpretation is left to the user’s professional judgement, e.g., using qualitative pedigree matrices^45^.

Considering sub-national coverage in general, the sub-national dataset for GDP covers 82 countries, representing 85% of the global population and producing 92% of global total GDP (PPP) in 2015 (Fig. 5). HDI sub-national data covers 39 countries and 66% of global population in 2015 (Fig. 6).

**Figure 7:**
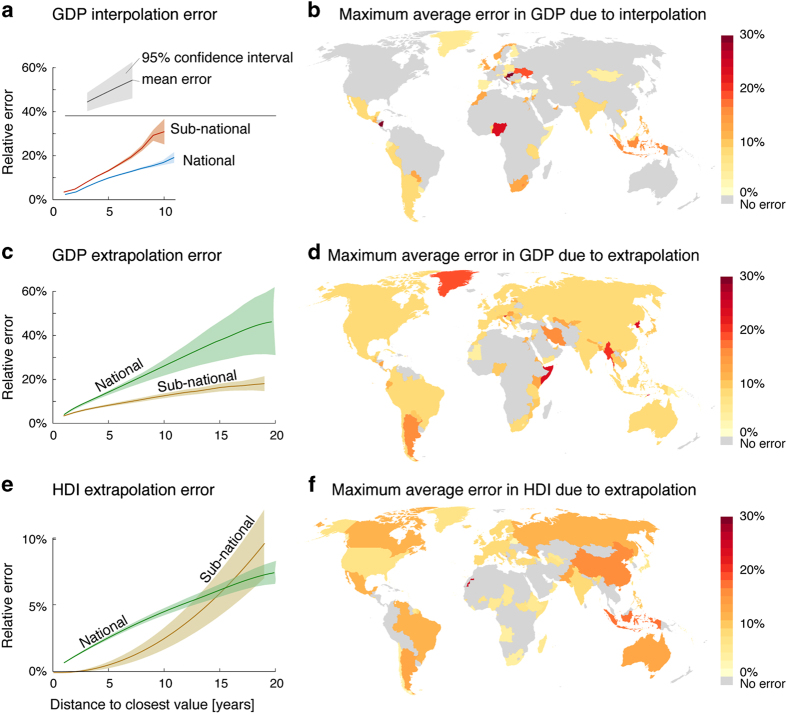
Error estimates originating from interpolation and extrapolation of missing data entries in the case of national and sub-national data. The error varies from year to year and we map here the maximum average error for each administrative area (either national or sub-national), which is estimated using the average of the global error analysis. The maximum temporal distance (in years) to the closest observed value is identified for each grid cell and the corresponding maximum average error is plotted. The error is therefore only indicative. *Note: different scale in GDP and HDI plots; and no interpolation was needed for HDI dataset, and thus, results are not available*.

In Fig. 7, the results for interpolation and extrapolation error estimations are given. As expected, relative error increases with increasing distance from observed data. The error for GDP interpolation at national scale is relatively small (<20% at 10 time steps distance) and consistent (narrow confidence interval), while for sub-national interpolation the error is somewhat larger but still rather consistent (Fig. 7a). This suggests that in the dataset analysed, reported GDP consistently varies sufficiently smoothly to be captured by a thin plate spline. In contrast, GDP extrapolation at national scale has slightly larger (ca. 25% at 10 time steps) and more variable errors, reflecting variability between countries within regions. Within countries, between the sub-national units, the extrapolation error is much smaller, reaching ca. 10% at 10 time steps (Fig. 7c). For HDI, extrapolation at national scale has much lower errors (ca. 4% at 10 time steps), suggesting greater homogeneity between countries (Fig. 7e). Extrapolation at sub-national scale has a different shape to the national one, resulting in smaller errors than national extrapolation at shorter distances (<10 time steps) and larger when more than 15 time steps away (Fig. 7e). The spatial distribution of maximum errors (Fig. 7b,d,f) is determined by the maximum distance to the closest value in each administrative area. As we can only quantify error in interpolation and extrapolation, and not due to other sources such as input data, no estimate is available for the many areas where data was complete.

Due to the shortage of sub-national HDI data over time, we used the same HDI ratio for all the years, i.e., we assumed that the HDI distribution within a country does not change. For some countries (Brazil, Canada, Chile and China), sub-national data were found for multiple years, which allowed us to i) estimate the subnational extrapolation error (Fig. 7e) and ii) evaluate how well our assumptions holds in these countries. To test the assumption, we used Pearson correlation (R_P_) to calculate the similarity between the years, i.e., we calculated how well the sub-national HDI ratios of two years, compared to that year’s national average, correlated with each other. Results below are all statistically highly significant (*P*<0.001):

- *Brazil*: for sub-national HDI ratios between years 2010 and 2000 R_P_=0.98, while it was lower between 2010 and 1990 (R_P_=0.94)
- *Canada*: for sub-national HDI ratios between years 2011 and 2005 R_P_=0.99, while it was almost as good between 2011 and 2000 (R_P_=0.985)
- *Chile*: for sub-national HDI ratios between years 2003 and 1990 R_P_=0.92
- *China*: for sub-national HDI ratios between years 2014 and 2003 R_P_=0.97, while it was lower between 2014 and 1997 (R_P_=0.90).

These findings show that for some countries, the spatial distribution of HDI within a country was temporally constant for the past decade, while over time the difference increased in Brazil and China. This should be considered when data are used.

## Usage Notes

**Figure 8:**
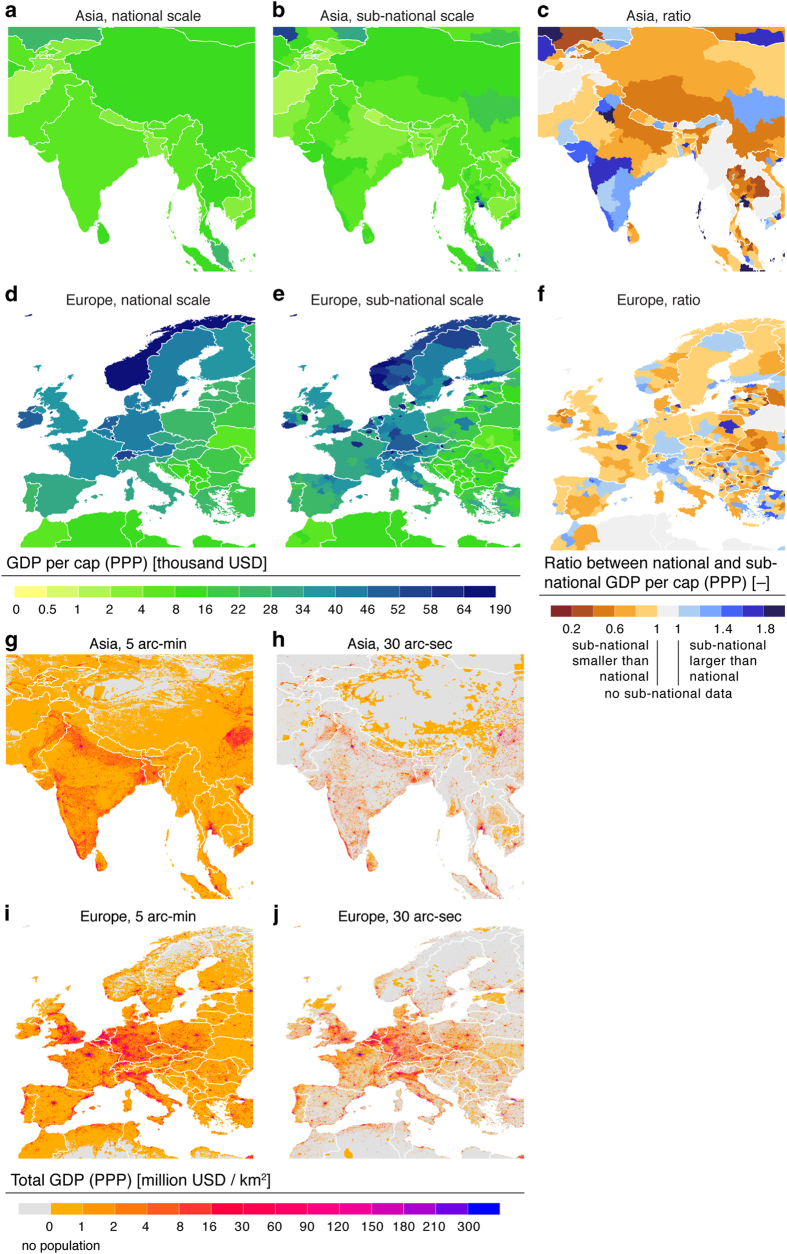
Examples of how resolution of data impacts on GDP (PPP) data products. First example shows of how our GDP (PPP) per capita product (**b**, **e**), based on sub-national data whenever available, differs from the conventionally used national data (**a**, **d**), in Asia (**a**, **b**) and Europe (**d**, **e**). The ratios between these two data products are shown in (**c**, **f**). Second example illustrates how the resolution of population data (5 arc-min, ~10 km at equator versus 30 arc-sec, ~1 km at equator) impacts on total gross domestic production (GDP) (PPP) data in Asia (**g**, **h**) and Europe (**i**, **j**). See population data sources in Table 2.

To support the usage of the data, we provide two examples to users illustrating how the scale and resolution of input data affects the product. In the first example, we illustrate how the conventionally used national data differs from our product, which is based on sub-national data whenever possible (Fig. 8a–f). In the second example, we illustrate how the resolution of the population data impacts on total GDP (PPP) product at grid scale (Fig. 8g–j). In both examples detailed maps are provided for two geographical areas, namely Asia and Europe.

Consistent with existing practices in the use of global GDP and HDI products, there are a several issues that users should be aware of when applying the datasets:

- Pedigree and associated accuracy of the dataset varies between regions and from year to year. To provide transparency on this issue, we report the source of the data, and possible data filling method of each time step in pedigree datasets.
- GDP per capita (PPP) and HDI represent the average value of an administrative unit in question and the dataset does not capture the possible heterogeneity within that administrative unit. In most cases, the administrative units are larger than those used in the G-Econ database^27,31^, which is therefore potentially more accurate.
- HDI sub-national data were available only for a single year (around the year 2010, see Supplementary Information), and they were used to scale the national data for each time-step. Thus, possible changes in sub-national HDI in relation to national HDI are not captured in this dataset.
- Estimates of error in interpolation and extrapolation are provided only as general indications of level of confidence in the data. Interested users may want to experiment with other interpolation or extrapolation methods (see pedigree layers for the time steps affected), but should bear in mind that the source data also has unknown uncertainty (see next point).
- We do not provide full estimates of uncertainty, only estimates of the accuracy of our interpolation and extrapolation methods. This is due to: i) uncertainty in the original reported input data is not available and the potentially volatile nature of GDP and HDI prevents estimation of reliable bounds (e.g., as a result of sudden economic shocks), and ii) we have a very poor understanding of how official statistics are generated (for every source, globally), and the errors involved are too complex to adequately capture with statistical methods without that information. Therefore, when using the dataset, implications of the underlying uncertainty of our data for analyses should be discussed qualitatively, just as would be done if the official data used in preparation of this dataset were used directly.

As indicated in the Introduction, the datasets are intended to replace the use of traditional country-scale data, such as for integrated modelling, hazard exposure and vulnerability analysis. Our data are based on up-to-date and best available estimates of areal averages and thus provide a valuable contribution to the scientific community working on global issues. Our results highlight the necessity of using high-resolution data with more representative information about the spatial variability.

## Additional information

**How to cite this article:** Kummu, M. *et al.* Gridded global datasets for Gross Domestic Product and Human Development Index over 1990–2015. *Sci. Data* 5:180004 doi: 10.1038/sdata.2018.4 (2018).

**Publisher’s note:** Springer Nature remains neutral with regard to jurisdictional claims in published maps and institutional affiliations.

## Supplementary Material



Supplementary Information

## Figures and Tables

**Table 1:** List of introduced development indicator datasets with their spatial extent, resolution and temporal extent.

**Dataset**	**Description**	**Spatial extent and resolution**	**Temporal extent**
*GDP per capita (PPP)*	Gross Domestic Production per capita (purchasing power parity), in constant 2011 international USD	Global; 5 arc-min; WGS84 projection	Annual; for each year over 1990–2015
*GDP (PPP)* *	Gross Domestic Production (purchasing power parity), in constant 2011 international USD	Global; 5 arc-min, 30 arc-sec; WGS84 projection	*5 arc-min*: Annual; for each year over 1990–2015. *30 arc-sec*: Annual; for years 1990, 2000, 2015
*HDI*	Human Development Index, based on method introduced 2010 and updated 2011. Dimensionless indicator between 0 and 1.	Global; 5 arc-min; WGS84 projection	Annual; for each year over 1990–2015

*Derived from GDP per capita (PPP) by multiplying it with i) 5 arc-min annual population dataset HYDE 3.2^7^ and ii) 30 arc-sec population data Global Human Settlement (GHS)^1^.

**Table 2:** List of input data for GDP per capita (PPP) and GDP (PPP) datasets.

**Dataset**	**Description**	**Source**	**Notes**
*Sub-national GDP per capita (PPP)*	*Spatial resolution*: sub-national (i.e., province, state, etc.; depending on country in question)*Temporal resolution*: annual data for 1960–2010; country specific coverage*Unit*: constant 2005 international USD	Gennaioli, *et al.*^28^	See Gennaioli, *et al.*^28^ for more details about the countries included and temporal coverage within each country. See also pedigree data for GDP and Fig. 5.
*National GDP per capita (PPP)*	*Spatial resolution*: national*Temporal resolution*: annual data for 1990–2015, country-specific coverage but mostly data for each year is available*Unit*: constant 2011 international USD	World Bank^32^	See World Bank^32^ for more details about the countries included and temporal coverage within each country. See also pedigree data for GDP and Fig. 5.
*National GDP per capita (PPP) for small island nations*	*Spatial resolution*: national*Temporal resolution*: only individual year or few years of data*Unit*: constant 2015 international USD^1^	CIA^33^	World Bank dataset did not include data for all countries and thus, those were extracted from CIA^33^ and listed in Supplementary Information.
*Population count (5 arc-min)*	*Spatial resolution*: 5 arc-min*Temporal resolution*: 1990, 2000–2015.*Unit*: population per grid cell	HYDE 3.2 ^7^	Population dataset was used to calculate GDP (PPP) from GDP per capita (PPP). The years not available (1991–1999) were linearly interpolated at grid scale based on data from years 1990 and 2000.
*Population count (30 arc-sec)*	*Spatial resolution*: 30 arc-sec*Temporal resolution*: 1990, 2000, 2015.*Unit*: population per grid cell	Global Human Settlement (GHS)^1^	Population dataset was used to calculate GDP (PPP) from GDP per capita (PPP) for three years.

**Table 3:** List of input data for HDI dataset.

**Dataset**	**Description**	**Source**	**Notes**
*Sub-national HDI data for Europe*	*Spatial resolution*: sub-national at NUTS* levels*Temporal resolution*: year 2007*Unit*: —	Eurostat^41^	List of countries for which data is available is given in Supplementary Information.
*Sub-national HDI data for elsewhere*	*Spatial resolution*: sub-national (i.e., province, state, etc.; depending on country in question)*Temporal resolution*: varies around year 2010, depending on country in question*Unit*: —	Varying sources, see details in Supplementary Information	List of countries for which data is available is given in Supplementary Information.
*National HDI*	*Spatial resolution*: national*Temporal resolution*: annual data for years 1990–2015.*Unit*: —	UNDP^30^	See category ‘No data, regional average’ in Fig. 6 for countries for which no national data were available. For these, regional average values were used (see methods)

*NUTS stands for Nomenclature of territorial units for statistics and is used by EUROSTAT to report its data at sub-national level.

**Table 4:** List of provided data files with their format and dimensions.

**Dataset**	**Format**	**Dimensions**	**Note**
*GDP per capita (PPP)*	NetCDF-4	Lat: 2160Lon: 4320Timesteps: 26	Gridded GDP per capita, derived from a combination of sub-national and national datasets
*GDP (PPP)—5 arc-min*	NetCDF-4	Lat: 2160Lon: 4320Timesteps: 26	Total GDP (PPP) of each grid cell, derived from GDP per capita (PPP) which is multiplied by gridded population data HYDE 3.2
*GDP (PPP)—30 arc-sec*	NetCDF-4	Lat: 21600Lon: 43200Timesteps: 3	Total GDP (PPP) of each grid cell, derived from GDP per capita (PPP) which is multiplied by gridded population data GHS
*Pedigree of GDP data*	NetCDF-4	Lat: 2160Lon: 4320Timesteps: 26	Reports the scale (national, sub-national) and type (reported, interpolated, extrapolated) of each year of data
*HDI*	NetCDF-4	Lat: 2160Lon: 4320Timesteps: 26	Gridded HDI, derived from a combination of sub-national and national datasets
*Pedigree of HDI data*	NetCDF-4	Lat: 2160Lon: 4320Timesteps: 26	Reports the level (national, sub-national) and type (reported, interpolated, extrapolated) of each year of data
*Administrative units*	NetCDF-4	Lat: 2160Lon: 4320Products: 2	Represents the administrative units used for GDP per capita (PPP) and HDI. National admin units have id 1–999, sub-national ones 1001-
Lat stands for latitudes, Lon stands for longitudes, Timesteps stands for number of years of data. GDP stands for Gross Domestic Production, PPP stands for Purchasing Power Parity, HDI stands for Human Development Index. GHS stands for Global Human Settlement.			
