# Supplementary material for: Gridded global datasets for Gross Domestic Product and Human Development Index over 1990–2015
Source: Sci Data. 2018 Feb 6;5:180004. doi: 10.1038/sdata.2018.4 (PMC5800392; doi:10.1038/sdata.2018.4)
Supplement: Supplementary Information [file sdata20184-s2.pdf]

Supplement to
Kummu et al
Gridded global datasets for Gross Domestic Product (GDP) and Human Development Index (HDI) over 1990-2015
Scientific Data

In this supplementary, the following information is provided:

- Data sources are listed for the following data
  - National GDP per capita (PPP)
  - Sub-national GDP per capita (PPP)
  - National HDI
  - Sub-national HDI

- Tabulated error estimates for interpolation and extrapolation

National GDP per capita (PPP)

Majority of the national data is based on World Bank Development indicators database: http://data.worldbank.org/data-catalog/world-development-indicators

National GDP from other sources than World Bank development indicators database

Source:	**CIA World Factbook (partly downloaded from http://www.indexmundi.com)**	**Trends from CIA World Factbook used to fill missing values at the start of the study period**	**EUROSTAT**
Countries:	American Samoa	Haiti	Réunion
	Andorra	Libya	
	Aruba	Maldives	
	British Virgin Islands	Qatar	
	Cayman Islands	Sao Tome and Principe	
	Falkland Islands (Malvinas)		
	Faroe Islands		
	French Polynesia		
	Gibraltar		
	Greenland		
	Guam		
	Guyana		
	Isle of Man		
	Korea, Dem. People’s Rep.		
	Liechtenstein		
	Monaco		
	Nauru		
	New Caledonia		
	Northern Mariana Islands		
	San Marino		
	Somalia		
	St. Martin		
	Taiwan, China		
	Turks and Caicos Islands		
	Virgin Islands (U.S.)		

Sub-national GDP per capita (PPP)

Source for sub-national GDP:	Gennaioli, N., La Porta, R., Lopez-de-Silanes, F., and Shleifer, A.: Human Capital and Regional Development, The Quarterly Journal of Economics, 128, 105-164, 10.1093/qje/qjs050, 2013.

National HDI

National HDI is based on UNDP Human Development Report

UNDP: Human Development Reports database, United Nations Development Programme, 2016.
http://hdr.undp.org/en/data

For following non-UN member countries HDI data is based on:

Macau	Macau in Figures, 2016	http://www.dsec.gov.mo/getAttachment/3fcdb33a-9238-44ca-8e4b-ebdb9c868e8e/E_MN_PUB_2016_Y.aspx
Taiwan	Taiwan Directorate General of Budget	http://www.dgbas.gov.tw/public/Data/491716362790WG0X9I.pdf

For other non-UN member countries, HDI based on old calculation method was used to scale the regional average HDI

Source:	Hastings (2009)	https://web.archive.org/web/20111005100501/http://www.unescap.org/pdd/publications/workingpaper/wp_09_02.pdf

Sub-national HDI

Sources to sub-national HDI can be divided into two: Eurostat and others

Eurostat: http://ec.europa.eu/eurostat/data/data

**Country**	**Source**	**Year**
Austria	Eurostat	2007
Belgium	Eurostat	2007
Bulgaria	Eurostat	2007
Czech Republic	Eurostat	2007
Denmark	Eurostat	2007
Finland	Eurostat	2007
France	Eurostat	2007
Germany	Eurostat	2007
Greece	Eurostat	2007
Hungary	Eurostat	2007
Ireland	Eurostat	2007
Italy	Eurostat	2007
Netherlands	Eurostat	2007
Norway	Eurostat	2007
Poland	Eurostat	2007
Portugal	Eurostat	2007
Romania	Eurostat	2007
Slovakia	Eurostat	2007
Slovenia	Eurostat	2007
Spain	Eurostat	2007
Sweden	Eurostat	2007
United Kingdom	Eurostat	2007

Other:

**Country**	**Source**	**Year**	**Link**
Argentina	UNDP	2012	http://research.omicsgroup.org/index.php/List_of_Argentine_provinces_by_Human_Development_Index
Australia	Omicsgroup	N/A	http://research.omicsgroup.org/index.php/List_of_Australian_states_and_territories_by_Human_Development_Index
Brazil	UNDP, with IPEA and FJP: Human D	2010	http://research.omicsgroup.org/index.php/List_of_Brazilian_federative_units_by_Human_Development_Index
Canada	Hazell et al (2012): The Human Dev	2011	http://research.omicsgroup.org/index.php/List_of_Canadian_provinces_and_territories_by_Human_Development_Index
Chile	UNDP	2003	https://en.wikipedia.org/wiki/Ranked_lists_of_Chilean_regions#By_national_HDI
China	UNDP	2014	https://en.wikipedia.org/wiki/List_of_administrative_divisions_of_Greater_China_by_Human_Development_Index
El Salvador	UNDP	2008	https://en.wikipedia.org/wiki/List_of_Salvadoran_Departments_by_Human_Development_Index
India	Sharma et al (2011) India Human D	2008	https://en.wikipedia.org/wiki/List_of_Indian_states_and_territories_by_Human_Development_Index#cite_note-report2011-2
Indonesia	Badan Pusat Statistik of Indonesia	2015	https://en.wikipedia.org/wiki/List_of_Indonesian_provinces_by_Human_Development_Index
Japan	Tashi Choden et al (2007) Gross Na	2000	https://en.wikipedia.org/wiki/List_of_Japanese_prefectures_by_Human_Development_Index
Mexico	UNDP (2010):El Indice de Desarroll	2010	http://research.omicsgroup.org/index.php/List_of_Mexican_states_by_Human_Development_Index
Pakistan	SPDC research report	2012	https://en.wikipedia.org/wiki/List_of_districts_of_Pakistan_by_Human_Development_Index
Philippines	Philippine Human Development Ne	2008/2009	http://research.omicsgroup.org/index.php/List_of_Philippine_provinces_by_Human_Development_Index
Russia	UNDP	2010	https://en.wikipedia.org/wiki/List_of_federal_subjects_of_Russia_by_Human_Development_Index
South Africa	UNDP	2003	http://research.omicsgroup.org/index.php/List_of_South_African_provinces_by_Human_Development_Index
USA	Originally from American Human D	2006	http://mapscroll.blogspot.fi/2009/05/human-development-index-by-state.html
Venezuela	Venezuelan National Institute of St	N/A	http://research.omicsgroup.org/index.php/Ranked_list_of_Venezuelan_states#By_human_development_index

ERROR ESTIMATES FOR INTERPOLATION AND EXTRAPOLATION - NATIONAL

GDP (PPP) per capita - Gross Domestic Product (PPP) per capita

Distance from closest observed value (years)

	1	2	3	4	5	6	7	8	9	10	11	12	13	14	15	16	17	18	19	20	21	22	23
INTERPOLATION																							
high	2.49%	3.56%	6.01%	8.19%	10.13%	11.66%	13.09%	14.70%	16.10%	17.95%	21.57%												
mean	2.33%	3.49%	5.89%	8.00%	9.87%	11.34%	12.70%	14.17%	15.38%	16.84%	19.14%												
low	2.17%	3.42%	5.76%	7.80%	9.61%	11.02%	12.30%	13.65%	14.65%	15.74%	16.71%												
EXTRAPOLATION																							
high	4.24%	7.41%	10.24%	12.94%	15.59%	18.35%	21.18%	24.02%	27.10%	30.40%	33.87%	37.22%	40.73%	44.25%	47.64%	51.07%	55.06%	58.43%	61.03%	64.55%	70.27%	78.12%	86.02%
mean	4.05%	7.07%	9.73%	12.22%	14.60%	16.98%	19.36%	21.70%	24.14%	26.64%	29.20%	31.66%	34.15%	36.51%	38.87%	41.22%	43.87%	45.96%	47.47%	49.20%	51.62%	53.92%	53.74%
low	3.85%	6.73%	9.21%	11.50%	13.62%	15.61%	17.54%	19.37%	21.19%	22.89%	24.53%	26.11%	27.57%	28.77%	30.10%	31.38%	32.68%	33.49%	33.90%	33.85%	32.96%	29.72%	21.47%

HDI - Human Development Index

Distance from closest observed value (years)

	1	2	3	4	5	6	7	8	9	10	11	12	13	14	15	16	17	18	19	20	21	22	23
EXTRAPOLATION																							
high	0.71%	1.25%	1.75%	2.23%	2.69%	3.14%	3.57%	3.99%	4.39%	4.77%	5.13%	5.51%	5.85%	6.20%	6.56%	6.94%	7.32%	7.70%	8.03%	8.29%	8.61%	8.93%	9.59%
mean	0.68%	1.19%	1.67%	2.12%	2.57%	2.99%	3.40%	3.79%	4.16%	4.51%	4.83%	5.17%	5.47%	5.78%	6.09%	6.41%	6.72%	7.02%	7.26%	7.44%	7.62%	7.74%	7.91%
low	0.65%	1.14%	1.59%	2.02%	2.44%	2.84%	3.22%	3.58%	3.92%	4.24%	4.53%	4.83%	5.09%	5.35%	5.62%	5.88%	6.13%	6.35%	6.50%	6.58%	6.64%	6.55%	6.24%

Note: for HDI no interpolation was needed; see main text.

low - high values represents the 95% confidence interval

ERROR ESTIMATES FOR INTERPOLATION AND EXTRAPOLATION - SUB-NATIONAL

GDP (PPP) per capita - Gross Domestic Product (PPP) per capita

	**Distance from closest observed value (years)**																						
	1	2	3	4	5	6	7	8	9	10	11	12	13	14	15	16	17	18	19	20	21	22	23
INTERPOLATION																							
high	3.72%	4.97%	8.04%	11.00%	13.58%	16.80%	20.19%	24.75%	31.67%	36.66%													
mean	3.51%	4.87%	7.88%	10.74%	13.22%	16.24%	19.37%	23.48%	29.23%	30.91%													
low	3.30%	4.78%	7.72%	10.48%	12.85%	15.69%	18.54%	22.21%	26.80%	25.16%													
EXTRAPOLATION																							
high	3.54%	5.29%	6.63%	7.70%	8.71%	9.70%	10.68%	11.63%	12.69%	13.59%	14.51%	15.43%	16.05%	16.99%	18.02%	18.75%	19.26%	20.27%	21.42%				
mean	3.32%	4.94%	6.20%	7.22%	8.16%	9.04%	9.90%	10.77%	11.70%	12.51%	13.33%	14.10%	14.67%	15.46%	16.27%	16.80%	17.11%	17.63%	18.06%				
low	3.10%	4.59%	5.77%	6.74%	7.60%	8.38%	9.13%	9.91%	10.71%	11.43%	12.15%	12.78%	13.28%	13.93%	14.52%	14.85%	14.95%	15.00%	14.69%				

HDI - Human Development Index

	**Distance from closest observed value (years)**																									
	1	2	3	4	5	6	7	8	9	10	11	12	13	14	15	16	17	18	19	20	21	22	23	24	25	26
EXTRAPOLATION																										
high	0.00%	0.05%	0.22%	0.46%	0.77%	1.14%	1.59%	2.09%	2.67%	3.32%	4.03%	4.81%	5.65%	6.56%	7.55%	8.59%	9.71%	10.89%	12.14%	13.46%	14.84%	16.30%	17.82%	19.40%	21.06%	22.78%
mean	0.00%	0.01%	0.13%	0.30%	0.52%	0.81%	1.15%	1.54%	1.99%	2.50%	3.07%	3.68%	4.36%	5.09%	5.88%	6.72%	7.62%	8.57%	9.58%	10.65%	11.77%	12.95%	14.18%	15.47%	16.82%	18.22%
low	0.00%	0.00%	0.03%	0.13%	0.28%	0.47%	0.71%	0.99%	1.32%	1.69%	2.10%	2.56%	3.07%	3.61%	4.21%	4.84%	5.53%	6.25%	7.02%	7.84%	8.69%	9.60%	10.55%	11.54%	12.57%	13.66%

Note: for HDI no interpolation was needed; see main text.

low - high values represents the 95% confidence interval
